# Commentary: Children's Sense of Fairness as Equal Respect

**DOI:** 10.3389/fpsyg.2020.00107

**Published:** 2020-01-31

**Authors:** Luca Surian, Francesco Margoni

**Affiliations:** Department of Psychology and Cognitive Sciences, University of Trento, Trento, Italy

**Keywords:** infants, fairness, moral judgment, distributive justice, cooperation

Engelmann and Tomasello (2019) (henceforth E&T) propose a model of the sense of fairness in humans that makes interesting evolutionary and ontogenetic claims. The need for collaboration, they say, was a major evolutionary selective force and children's sense of fairness is not centered on the material distributions of resources *per se* (McAuliffe et al., 2017), but on the social meaning of such distributions. Our sense of fairness is best understood as sensitivity to interpersonal respect that appears, at the age of 3 years, in children's distributive actions. The supporting evidence is that even preschoolers can display aversion to advantageous inequality, but they do so only if they are first involved in a collaborative activity. Preschoolers accept unequal distributions as long as these result from fair procedures (Shaw and Olson, 2014), or are based on merit. We would like to raise three problems for their ontogenetic view and, more specifically, with their claims that “interdependent collaborative activities represent the key interactive context for children's developing sense of fairness” (p. 456) and that “children display an aversion to inequity (…) at about age 3, in (and only in) interdependent collaborative activities” (p. 462). Our first point is a methodological issue about what is relevant evidence for developmental models of fairness, the second concerns some experimental results that challenge their account, and the third is a reflection on their concept of *respect*.

First, when we aim at evaluating theories on the ontogenesis of the sense of fairness in humans, why should we focus exclusively, like E&T do, on data obtained from tasks in which preschoolers and older children are asked explicitly to perform or judge distributive actions? For a few decades, in the research on moral development, many researchers considered children's justifications and verbal moral reasoning as more important than their non-verbal choices and actions. It would be a pity if, after having overcome this bias, the field now falls into a new tendency to narrow its focus on responses to economic games as the primary, if not the only, relevant empirical evidence. We suggest that it is important to look also at measures of evaluative processes obtained from implicit tasks such as those used in the infant literature. Infants' looking behavior and manual responses, despite the difficulty of interpreting their meaning, have been successfully employed in investigating the origins of core concepts in several domains, including naïve physics, mathematics, and mental state reasoning (Carey, 2009). There is no reason to ignore this kind of evidence when we study the origins of moral cognition and the sense of fairness (Hamlin et al., 2007).

There is now abundant evidence showing that infants generate expectations about resource distributions, and, quite likely, evaluate distributive actions (Schmidt and Sommerville, 2011; Sloane et al., 2012; DesChamps et al., 2016; Meristo et al., 2016; Surian and Franchin, 2017; Margoni and Surian, 2018; Buyukozer Dawkins et al., 2019). This evidence is highly relevant to any claim on the ontogenetic origins of fairness in humans. Infant data consist mainly of spontaneous looking times at events that conform or violate egalitarian principles of fairness, as well as merit-based principles. These responses have repeatedly shown that infants in their first year of life expect a distributor to distribute windfall resources equally among similar recipients (Meristo et al., 2016; Buyukozer Dawkins et al., 2019). Older infants, by 20–24 months, expect distributions to be consistent with relative merit (Sloane et al., 2012; Surian and Franchin, 2017). Infant studies have also looked at manual choices and consistently found that infants prefer fair over unfair individuals (Margoni and Surian, 2018). Moreover, infants' looking times correlate with their spontaneous sharing behavior (Schmidt and Sommerville, 2011) and reveal that infants link distributive behaviors to rewards and punishments, as well as to admonishments and praises delivered toward fair or unfair distributors (DesChamps et al., 2016). Overall, this evidence strongly suggests that a sense of fairness emerges well before the third birthday and does not require any involvement in collaboration to be triggered. These data challenge classical views that deny the role of domain-specific adaptations and provide support for accounts of the origins of the sense of fairness that emphasize evolutionary adaptations rather than social learning.

According to E&T, children's sense of fairness relies on their sensitivity to interpersonal respect. They say that their account “can explain why children care about partiality and impartiality in the first place: it is precisely that the person being impartial in distributing resources signals respect to everyone as equal participants” (p. 461). In their account, however, it is not clear whether and how the child represents the concept of respect. This needs to be clarified in future work if one wants to argue, following E&T, that while impartiality in distributive actions is a crucial cue that children and adults use to infer respect, or lack of it, respect goes, in essence, well beyond procedural impartiality. Moreover, it is not clear how the notion of equal respect is related to other forms of respect, such as those found in social relations that are asymmetrical with regard to power. It seems that the concept of interpersonal respect on which E&T center their model works well only when collaborations among peers are involved. However, respect is also a fundamental aspect of hierarchical relations. A concept of mutual respect among equals may thus fall short of covering other widespread social relations, such as those involving dominant and subordinate individuals, relations that are understood by the second year of life (Thomsen et al., 2011; Margoni et al., 2018).

Once we consider a broad range of evidence, it seems that an incipient sense of fairness emerges in the first 2 years of life and its emergence does not depend on collaborations with peers or adults. Research in infants shows that the sense of fairness does not need collaborative work to emerge, but collaborative work may be a key factor in allowing children to flexibly employ it in a variety of tasks and contexts, including tasks that require first party evaluations, rely on explicit processing and use more active responses than those used in the infant literature. This conclusion, while running against some aspects of E&T's model, is consistent with their evolutionary claims and the idea that respect plays a crucial role in how older children understand fairness in distributive justice and why they care about it.

## Author Contributions

All authors listed have made a substantial, direct and intellectual contribution to the work, and approved it for publication.

### Conflict of Interest

The authors declare that the research was conducted in the absence of any commercial or financial relationships that could be construed as a potential conflict of interest.
